# Worldwide prevalence of heart failure due to congenital heart disease: An analysis from the Global Burden of Disease Study 2021

**DOI:** 10.1016/j.ijcchd.2024.100552

**Published:** 2024-11-16

**Authors:** Ahmed Kheiwa, Inhae Baek, Ian S. Harris, Abdul Mannan Khan Minhas, Dmitry Abramov

**Affiliations:** a Department of Medicine, Division of Cardiology, Adult Congenital Heart Disease Program, Loma Linda University, Loma Linda, CA, USA; b Department of Medicine, Loma Linda University, Loma Linda, CA, USA; c Department of Medicine, Division of Cardiology, University of California, San Francisco, CA, USA; d Department of Medicine and Section for Cardiovascular Research, Baylor College of Medicine, Houston, TX, USA; e Department of Medicine, Division of Cardiology, Loma Linda University, Loma Linda, CA, USA

**Keywords:** Heart failure, Congenital heart disease, Global burden of disease

## Abstract

**Background:**

There are limited data on the prevalence of heart failure (HF) due to congenital heart diseases (CHD).

**Methods:**

The Global Burden of Disease (GBD) dataset was used to analyze the disease burden of HF due to CHD between 1990 and 2021. Age-standardized rates (ASR) (crude prevalence rates for age groups) and total percent change for the overall worldwide burden as well as among subgroups based on age and geographic regions were determined.

**Results:**

The global prevalence of HF due to CHD in 1990 and 2021 was 2,494,547 (95 % IU 2,054,729 to 3,030,909) and 3,155,991 (95 % IU 2,578,552 to 3,843,062) individuals respectively, with an increase in ASR from 41.02 (33.79–49.79) to 45.33 (37.15–55.17) per 100,000 individuals during that period. HF due to CHD in 2021 was most common in children aged 2–4, crude rate of 248.44 (195.99–302.57), followed by children <1 years of age, crude rate of 142.75 (116.87–174.26), and children aged 5–14, crude rate of 89.55 (62.35–129.61). During the study period, there was an increase in the prevalence of HF due to CHD among most age groups, other than children <1 year of age who had stable rates and individuals >70 who had no reported prevalence. There were geographic differences in the prevalence and trends of HF due to CHD.

**Conclusions:**

There are differences in prevalence and trends of HF due to CHD among age groups and worldwide regions. These results demonstrate the worldwide burden and trends of HF due to CHD.

## CRediT authorship contribution statement

**Ahmed Kheiwa:** Writing – review & editing, Writing – original draft. **Inhae Baek:** Writing – review & editing. **Ian S. Harris:** Writing – review & editing. **Abdul Mannan Khan Minhas:** Writing – review & editing, Visualization, Methodology, Formal analysis. **Dmitry Abramov:** Writing – review & editing, Writing – original draft, Supervision, Project administration, Conceptualization.

## Disclosures

No relevant disclosures.

Recent advances in surgical techniques and post-operative care have led to significant improvement in congenital heart disease (CHD) outcomes and allowed most children with CHD in developed countries to survive to adulthood. Currently, the number of adults with CHD in developed countries exceeds the number of children with CHD [1,2]. Despite improvement in early mortality, CHD patients encounter cumulative complications over their lifespan related to detrimental effects of cyanosis, residual shunts, valvular dysfunction, volume and pressure overload, arrhythmias, and perhaps intrinsic, cell-autonomous myocardial defects related to the underlying developmental abnormality [[3], [4], [5]]. These residual hemodynamic and electrophysiological abnormalities often result in heart failure (HF) which is associated with significant morbidity and mortality. Although the prevalence of CHD in the US and worldwide has been well described [[6], [7], [8], [9], [10]], there are limited data on the prevalence of HF in individuals with CHD [11,12]. Herein, we present data on the global burden of HF due to CHD in a geographically wide and inclusive spectrum of countries and territories from the Global Burden of Disease (GBD) dataset. These data may have important implications for global public health efforts to understand the burden and potentially to allocate resources for screening and management strategies for patients with CHD and particularly for patients with HF due to CHD.

The data in this study were extracted from the GBD 2021 dataset [13,14]. Detailed methodology for the GBD, particularly as it relates to the estimation of the burden of CHD have been previously described [10]. Briefly, the GBD assessed the prevalence CHD epidemiology based on systematic literature review, congenital birth defect registries, and administrative data (based on ICD codes). Heart Failure estimates within the GBD were subsequently based on available data using proportional allocation (for further information see Supplementary material available in reference 10). GBD was used to analyze the disease burden of HF due to CHD between 1990 and 2021. The prevalence of cases and their 95 % uncertainty intervals (UIs) and age‐standardized rate (ASR) per 100,000 (crude prevalence rates for age groups) were determined by age, sex, year, socio-demographic index (SDI), regions, and countries and territories as characterized within the GBD. The SDI within the GBD is a composite indicator of country development status based on total fertility rate for females under age 25, mean education for individuals aged 15 and over, and lag-distributed income per capita. The temporal trends from 1990 to 2021 were described using total percent change (TPC) and corresponding 95 % uncertainty interval (95 % UI) for global population and subgroups. TPC was considered to be declining if the TPC was negative and the 95 % UI excluded 0 and considered to be increasing if the TPC was positive and the 95 % UI excluded 0. Prevalence, ASRs, and TPCs were extracted from GBD 2021 dataset [13].

In the GBD, the global prevalence of CHD irrespective of HF in 1990 and 2021 was 11,787,061 (95 % IU: 10,500,375–13,054,728) and 15,774,457 (95 % IU: 14,041,229–17,389,520) individuals respectively, with no change in ASR from 209.53 (95 % IU: 186.40–231.496) to 210.70 (95 % IU: 187.92–232.48). The global prevalence of HF due to CHD in 1990 and 2021 was 2,494,547 (95 % UI: 2,054,729–3,030,909) and 3,155,991 (95 % UI: 2,578,552 -3,843,062) individuals respectively. There was an increase in ASR from 41.02 (33.79–49.79) to 45.33 (37.15–55.17) per 100,000 individuals during that period, TPC of 0.11 (0.07–0.14). (Table 1). The overall prevalence of HF due to CHD is shown in Supplementary Table 1. The rates of CHD irrespective of HF, rates of HF due to CHD, and the rates of HF due to any cause by age range in 2021 for comparison are shown in Supplementary Table 2.

**Table 1:** Age Standardized Rates (ASR) and Total Percent Change (TPC) of Heart Failure due to Congenital Heart Disease.

	1990 ASR per 100,000 (95 % UI)	2021 ASR per 100,000 (95 % UI)	TPC of ASR (1990–2021) (95 % UI)
Global	41.02 (33.79–49.79)	45.33 (37.15–55.17)	0.11 (0.07–0.14)
**Age groups**^a^			
<1 years	143.58 (118.15–175.45)	142.75 (116.87–174.26)	−0.01 (−0.03 to 0.02)
2–4 years	232.19 (184.45–281.98)	248.44 (195.99–302.57)	0.07 (0.04–0.1)
5–14 years	80.7 (56.87–115.98)	89.55 (62.35–129.61)	0.11 (0.05–0.17)
15–49 years	8.71 (6.44–11.53)	10.02 (7.39–13.39)	0.15 (0.07–0.25)
50–69 years	1.4 (1.01–1.93)	2.06 (1.43–2.87)	0.47 (0.32–0.63)
>70 years	0 (0–0)	0 (0–0)	0 (0–0)
**Sex**			
Male	44.6 (36.67–54.27)	48.13 (39.32–58.66)	0.08 (0.04–0.11)
Female	37.22 (30.74–45.36)	42.33 (34.75–51.63)	0.14 (0.1–0.17)
**SDI**			
High SDI	67.22 (55.38–81.44)	67.22 (55.38–81.44)	0.01 (−0.03 to 0.05)
High-middle SDI	50.91 (42.09–62.19)	58.63 (48.5–71.19)	0.15 (0.11–0.19)
Middle SDI	38.58 (31.93–46.65)	48.68 (40.5–58.88)	0.26 (0.22–0.3)
Low-Middle SDI	34.02 (27.86–41.57)	40.04 (32.55–48.85)	0.18 (0.14–0.22)
Low SDI	26.95 (21.04–34.12)	32.05 (24.77–40.86)	0.19 (0.15–0.23)
**Regions**			
Andean Latin America	48.88 (38.91–61.06)	55.96 (45.09–69.79)	0.14 (0.08–0.21)
Australasia	61.99 (50.61–74.87)	65.86 (53.26–81.47)	0.06 (−0.01 to 0.14)
Caribbean	50.47 (41.17–62.32)	43.46 (35.14–54.02)	−0.14 (−0.18 to −0.1)
Central Asia	48.42 (38.23–60.84)	58.41 (46.15–73.68)	0.21 (0.16–0.26)
Central Europe	80.68 (66.17–99.88)	89.14 (73.02–109.45)	0.1 (0.06–0.15)
Central Latin America	51.6 (42.45–62.92)	65.99 (54.59–80.15)	0.28 (0.22–0.34)
Central Sub-Saharan Africa	24.22 (17.46–32.29)	31.02 (22.6–41.83)	0.28 (0.2–0.36)
East Asia	34.52 (28.79–41.8)	51.41 (42.83–61.94)	0.49 (0.42–0.56)
Eastern Europe	70.8 (58.08–85.84)	67.93 (55.08–83.44)	−0.04 (−0.09 to 0.01)
Eastern Sub-Saharan Africa	24.2 (17.96–31.82)	27.53 (20.32–36.48)	0.14 (0.1–0.18)
High-income Asia Pacific	64.34 (52.71–78.96)	68.46 (55.67–82.84)	0.06 (0.01–0.12)
High-income North America	65.92 (54.19–80.54)	61.01 (48.88–75.1)	−0.07 (−0.12 to −0.02)
North Africa and Middle East	52.4 (42.39–64.32)	55.88 (45.21–68.73)	0.07 (0.02–0.11)
Oceania	26.55 (21.3–33.16)	25.66 (20.37–32.04)	−0.03 (−0.09 to 0.02)
South Asia	33.91 (28.19–40.98)	40.28 (33.11–48.87)	0.19 (0.15–0.22)
Southeast Asia	30.97 (25.26–37.5)	34.16 (28.02–41.49)	0.1 (0.07–0.14)
Southern Latin America	45.14 (35.95–56.63)	55.94 (44.94–69.52)	0.24 (0.15–0.33)
Southern Sub-Saharan Africa	28.78 (22.2–36.21)	36.05 (27.64–45.43)	0.25 (0.2–0.3)
Tropical Latin America	41.72 (34.06–49.84)	49.92 (40.46–60.72)	0.2 (0.14–0.26)
Western Europe	68.45 (56.61–83.29)	70.5 (57.61–85.89)	0.03 (−0.01 to 0.07)
Western Sub-Saharan Africa	29.92 (22.3–38.61)	36.31 (27.38–47.61)	0.21 (0.16–0.27)

SDI: socio-demographic index.

a Age groups represent crude prevalence rate per 100,000. UI: uncertainty interval.

In 2021, the prevalence per 100,000 of HF due to CHD was highest in children aged 2–4 (248.44, 195.99 to 302.57) followed by < 1 years of age 142.75 (116.87–174.26) and 5–14 years of age 89.55 (62.35–129.61). There was a stable trend for burden of HF due to CHD between 1990 and 2021 among children age groups of <1years TPC (−0.01 %, −0.03 to 0.02 %), but an increase for the remaining age groups up to age 70 (Table 1). The TPC was 0.07 (0.04–0.1) for children aged 2–4, 0.11 (0.05–0.17) for children aged 5–15, 0.11 (0.05–0.17) for adolescents and young adults aged 15–49, and TPC was 0.47 (0.32–0.63) for older adults aged 50–69.

**Fig. 1:**
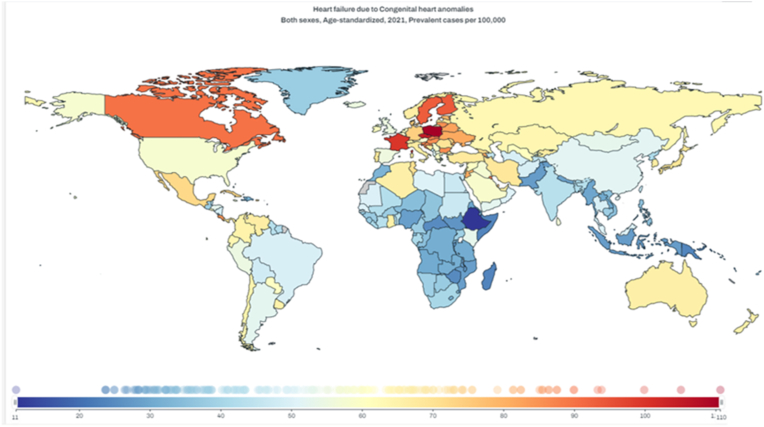
Age Standardized Prevalence Rate per 100,000 of heart failure due to congenital heart disease by countries and territories in 2021.

Among available geographic classifications in 2021, the highest ASR per 100,000 was in Central Europe 89.14 (73.02–109.45) while high-income North American and Western European regions had ASRs of 61.01 (48.88–75.1) and 70.5 (57.61–85.89) respectively. The geographic region with the lowest reported ASR was in Oceania at 25.66 (20.37–32.04). The prevalence and trends in other geographic regions, including as characterized by sociodemographic index (SDI) are shown in Table 1, with higher SDI regions generally having higher ASRs compared to lower SDI regions. Fig. 1 demonstrates the ASR of HF due to CHD in 2021 among countries. Country-specific 2021 prevalence is shown in Supplemental Table 3.

These data highlight the global burden of HF due to CHD. There are limited prior data on the prevalence and trends specifically of HF due to CHD, either in developed countries or worldwide. Previous reports have identified HF as the leading cause of death in CHD patients with a significant increase in HF-related hospital admissions over the past decade in the United States [15,16]. These hospitalization trends were particularly prominent in CHD patients with complex congenital lesions as they advance in age [[17], [18], [19], [20], [21]]. Expanding on previous reports on prevalence of CHD (irrespective of HF) and the prevalence of HF hospitalizations in patients with CHD, we report on the prevalence and trends of the global burden of HF due to CHD using geographically diverse data and data among cohorts based on age and sex.

We note that the prevalence of HF among younger patients due to CHD is greater than the burden among older age groups, which likely reflects a greater prevalence of overall CHD in the younger population. Additionally, HF due to CHD may be higher in younger individuals because HF in this population may be initially diagnosed in childhood with complex congenital lesions resulting in a poor prognosis once HF diagnosed, thus preventing young patients from surviving to older age. However, we also demonstrated an increasing trend in prevalence of HF due to CHD among most age groups, including adults. The increase in HF in older individuals may reflect both the natural history of CHD and better early CHD management, which lead to improved patient survival even if HF subsequently develops.

Additionally, we highlight potential geographic variation in prevalence of HF due to CHD. A greater prevalence was noted in regions with higher SDI and certain geographic regions compared to lower SDI regions. Regarding trends based on SDI, we observed that the highest SDI region demonstrated stable rates of HF due to CHD during the study period while remaining SDI regions demonstrated increases in HF due to CHD. There are several plausible explanations for these regional findings, including explanations related to the ascertainment/diagnosis and management of CHD. HF due to CHD may be under-recognized as underdiagnosis of CHD itself is common, particularly in developing countries, which may subsequently lead to early mortality from CHD without a HF diagnosis [20]. Alternatively, underdiagnosis and lack of appropriate early interventions for CHD in certain geographic or socioeconomic regions may subsequently increase the prevalence of HF associated with CHD, while prompt identification of CHD in high SDI regions that have specialized resources to optimize treatment may contribute to stable prevalence over of HF due to CHD over time. Further research will be needed to further clarify the potential reasons for differences in geographic prevalence and trends of HF associated with CHD, including to determine whether trends in HF may be primarily due to regional differences in diagnosis or differences in care including surgical intervention.

This study has limitations. The GBD collects data from various sources and applies statistical modeling, which may vary over time. Data sources and quality may vary by country or region which may lead to variability in data quality. Data about treatment are not available. The use of administrative data with ICD codes has known limitations for identifying true CHD [[22], [23], [24]]. Variability in HF diagnosis in the CHD population could be related to the fact that CHD patients may not present with the typical HF symptoms and different phenotypes of HF often present the clinical course which may not be reported as HF. Data on the prevalence of different HF severities in patients with CHD are not presented. We were unable to categorize the prevalence of HF due to CHD by specific etiologies of CHD. However, the strength of GBD data involves a consistent epidemiologic approach to prevalence tracking over time in a dataset that is commonly utilized for public health disease tracking.

In conclusion, we report on the worldwide prevalence and trends of HF attributable to CHD. We identified age and regional differences in prevalence and trends of HF due to CHD. Despite acknowledged limitations, these results have important implications for tracking the prevalence of HF as the common end-stage complication of CHD, which may facilitate regional and global efforts to improve the care and outcomes of the CHD population.

## Declaration of competing interest

The authors report no relationships that could be construed as a conflict of interest.
