# Supplementary material for: Worldwide prevalence of heart failure due to congenital heart disease: An analysis from the Global Burden of Disease Study 2021
Source: Int J Cardiol Congenit Heart Dis. 2024 Nov 16;19:100552. doi: 10.1016/j.ijcchd.2024.100552 (PMC11803120; doi:10.1016/j.ijcchd.2024.100552)
Supplement: Supplementary file 1 [file mmc1.docx]

Supplemental Table 1: Prevalence of Heart Failure due to Congenital Heart Disease. SDI: socio-demographic index

|  | 1990 Prevalence (95% UI) | 2021 Prevalence (95% UI) |
| --- | --- | --- |
| Global | 2494547 (2054729 to 3030909) | 3155991 (2578552 to 3843062) |
| **Age groups** |  |  |
| <1 years | 183417 (150938 to 224131) | 180862 (148068 to 220781) |
| 2-4 years | 236012 (174554 to 312542) | 395691 (291938 to 528676) |
| 5-14 years | 853477 (677999 to 1036468) | 1001390 (789962 to 1219567) |
| 15-49 years | 903226 (636492 to 1298022) | 1212230 (844079 to 1754493) |
| 50-69 years | 9575 (6861 to 13153) | 29601 (20524 to 41253) |
| >70 years | 0 (0 to 0) | 0 (0 to 0) |
| **Sex** |  |  |
| Male | 1394887 (1145516 to 1697325) | 1721751 (1400589 to 2096172) |
| Female | 1099660 (907746 to 1341545) | 1434240 (1179388 to 1749393) |
| **SDI** |  |  |
| High SDI | 445633 (369288 to 540857) | 421446 (348479 to 513370) |
| High-middle SDI | 490600 (405550 to 599890) | 478208 (397204 to 580609) |
| Middle SDI | 775841 (642578 to 939816) | 951616 (786755 to 1149428) |
| Low-Middle SDI | 561955 (458046 to 685125) | 795548 (643913 to 969681) |
| Low SDI | 217774 (169089 to 276639) | 506661 (389763 to 648334) |
| **Regions** |  |  |
| Andean Latin America | 24832 (19713 to 31092) | 35498 (28669 to 44108) |
| Australasia | 10098 (8285 to 12205) | 13369 (10824 to 16531) |
| Caribbean | 20261 (16562 to 24975) | 17455 (14136 to 21636) |
| Central Asia | 43536 (34602 to 54512) | 56979 (44942 to 71984) |
| Central Europe | 80779 (65842 to 100492) | 54891 (45017 to 67102) |
| Central Latin America | 114108 (93420 to 139277) | 146403 (121192 to 177334) |
| Central Sub-Saharan Africa | 22085 (15870 to 29517) | 62319 (45224 to 84014) |
| East Asia | 411963 (344508 to 499141) | 491725 (408356 to 591871) |
| Eastern Europe | 127404 (104119 to 154834) | 80749 (64932 to 100723) |
| Eastern Sub-Saharan Africa | 76519 (57212 to 99906) | 168146 (123227 to 223199) |
| High-income Asia Pacific | 77666 (63713 to 95890) | 56284 (46471 to 67892) |
| High-income North America | 148121 (122596 to 180041) | 142314 (113976 to 175431) |
| North Africa and Middle East | 256701 (206858 to 316112) | 352432 (284086 to 434360) |
| Oceania | 2509 (2014 to 3125) | 4659 (3695 to 5780) |
| South Asia | 513033 (425110 to 621942) | 691208 (566879 to 847151) |
| Southeast Asia | 180689 (146948 to 219643) | 201258 (165210 to 245044) |
| Southern Latin America | 23287 (18532 to 29233) | 27476 (21927 to 34293) |
| Southern Sub-Saharan Africa | 20830 (16042 to 26202) | 29693 (22758 to 37447) |
| Tropical Latin America | 74828 (60812 to 90519) | 88409 (72030 to 107626) |
| Western Europe | 172771 (143476 to 209242) | 171049 (141757 to 208402) |
| Western Sub-Saharan Africa | 92528 (68771 to 120828) | 263675 (196640 to 345663) |

Supplemental Table 2: Crude rates per 100,000 of CHD, Heart Failure from any cause, and Heart Failure due to CHD. HF: Heart Failure, CHD: Congenital Heart Disease. UI: Uncertainty Interval

|  | 2021 Crude Rates of CHD per 100,000 (95% UI) | 2021 Crude Rates of HF due to CHD per 100,000 (95%UI) | 2021 Crude Rates of HF due to any cause per 100,000 (95%UI) |
| --- | --- | --- | --- |
| Global* | 210.70 (187.92-262.61) | 45.33 (37.15 to 55.17) | 703.26 (620.89 to 809.02) |
| age groups |  |  |  |
| <1 years | 979.56 (844.03-1140.78) | 142.75 (116.87 to 174.26) | 197.52 (164.74 to 241.73) |
| 2-4 years | 505.19 (437.73-575.14) | 248.44 (195.99 to 302.57) | 400.3 (321.93 to 495.3) |
| 5-14 years | 227.58 (197.37-262.61) | 89.55 (62.35 to 129.61) | 203.03 (158.65 to 261.34) |
| 15-49 years | 150.60 (131.26-168.62) | 10.02 (7.39 to 13.39) | 178.5 (151.15 to 210.64) |
| 50-69 years | 135.00 (116.86-151.97) | 2.06 (1.43 to 2.87) | 956.04 (792.34 to 1148.05) |
| >70 years | 126.36 (107.46-146.35) | 0 (0 to 0) | 5988.99 (5092.01 to 7186.13) |

*Overall global rates are age-standardized

Supplemental Table 3:

ASPRs per 100 000 Population for Heart Failure due to Congenital heart anomalies in 204 countries and territories in 2019

| Location | Value | Lower | Upper |
| --- | --- | --- | --- |
| Jordan | 13.44 | 9.31 | 19.14 |
| Slovenia | 13.39 | 9.66 | 18.69 |
| Taiwan | 13.19 | 10.14 | 17.16 |
| Canada | 12.45 | 9.61 | 16.17 |
| Kuwait | 12.01 | 8.43 | 16.96 |
| Norway | 11.91 | 8.37 | 16.69 |
| Viet Nam | 11.45 | 7.89 | 16.4 |
| China | 11.35 | 7.99 | 15.67 |
| Lithuania | 10.79 | 7.47 | 15.61 |
| Poland | 10.75 | 7.3 | 15.29 |
| Belarus | 10.57 | 7.21 | 15.64 |
| Estonia | 10.51 | 7.18 | 15.08 |
| Palestine | 10.41 | 7.34 | 14.94 |
| Japan | 10.31 | 7.35 | 14.35 |
| Sri Lanka | 10.25 | 7.15 | 14.22 |
| Saudi Arabia | 10.11 | 7.59 | 13.81 |
| Mexico | 9.95 | 6.92 | 14.03 |
| Mauritius | 9.65 | 6.64 | 13.77 |
| Hungary | 9.6 | 6.72 | 13.51 |
| Cuba | 9.23 | 6.73 | 12.74 |
| Iceland | 9.01 | 6.45 | 12.48 |
| Denmark | 8.99 | 6.47 | 12.25 |
| Venezuela | 8.97 | 6.26 | 12.75 |
| Bermuda | 8.96 | 6.46 | 12.15 |
| Democratic People's Republic of Korea | 8.86 | 6.37 | 12.18 |
| Sweden | 8.85 | 6.38 | 12.19 |
| Lebanon | 8.73 | 6.14 | 12.1 |
| Malaysia | 8.69 | 6.32 | 11.8 |
| Czechia | 8.67 | 6.17 | 12.34 |
| Croatia | 8.67 | 6.21 | 12.11 |
| Italy | 8.65 | 6.07 | 12.18 |
| Northern Mariana Islands | 8.58 | 6.23 | 11.89 |
| Slovakia | 8.56 | 6.05 | 11.89 |
| Ukraine | 8.45 | 5.62 | 12.22 |
| Barbados | 8.42 | 5.99 | 11.85 |
| United Arab Emirates | 8.42 | 5.88 | 11.82 |
| Indonesia | 8.38 | 5.74 | 11.73 |
| Serbia | 8.34 | 5.74 | 11.95 |
| Qatar | 8.32 | 5.89 | 11.59 |
| Maldives | 8.24 | 5.9 | 11.51 |
| Tunisia | 8.23 | 5.79 | 11.44 |
| Latvia | 8.19 | 5.54 | 12.02 |
| Kenya | 8.15 | 5.42 | 11.83 |
| El Salvador | 8.03 | 5.52 | 11.67 |
| Seychelles | 7.99 | 5.61 | 11.3 |
| Turkey | 7.98 | 5.79 | 11.17 |
| Iran (Islamic Republic of) | 7.97 | 5.56 | 11.15 |
| Paraguay | 7.9 | 5.43 | 11.49 |
| Antigua and Barbuda | 7.88 | 5.64 | 10.88 |
| Trinidad and Tobago | 7.85 | 5.66 | 10.83 |
| Panama | 7.79 | 5.48 | 10.82 |
| Ireland | 7.78 | 5.52 | 10.74 |
| Switzerland | 7.76 | 5.54 | 10.81 |
| Thailand | 7.71 | 5.48 | 10.95 |
| Armenia | 7.69 | 5.57 | 10.7 |
| Greece | 7.67 | 5.17 | 11.35 |
| Republic of Moldova | 7.66 | 5.2 | 11.13 |
| Iraq | 7.64 | 5.46 | 10.86 |
| Myanmar | 7.62 | 5.45 | 10.74 |
| Bosnia and Herzegovina | 7.59 | 5.3 | 10.94 |
| Cambodia | 7.55 | 5.4 | 10.77 |
| Colombia | 7.55 | 5.37 | 10.47 |
| Philippines | 7.54 | 5.14 | 10.77 |
| France | 7.54 | 5.6 | 10.2 |
| Bulgaria | 7.46 | 5.06 | 10.86 |
| Romania | 7.44 | 4.99 | 10.89 |
| Peru | 7.41 | 5.32 | 10.2 |
| Puerto Rico | 7.38 | 5.28 | 10.23 |
| Algeria | 7.3 | 5.2 | 10.45 |
| Finland | 7.21 | 5.27 | 9.9 |
| Cabo Verde | 7.2 | 4.47 | 11.09 |
| Ecuador | 7.14 | 5 | 10.37 |
| Netherlands | 7.13 | 5.17 | 9.79 |
| Bangladesh | 7.12 | 5 | 10.09 |
| Uruguay | 7.07 | 5.03 | 9.9 |
| Timor-Leste | 7.04 | 5.06 | 9.86 |
| United Kingdom | 6.98 | 4.93 | 9.89 |
| Malta | 6.95 | 5.03 | 9.55 |
| Tonga | 6.93 | 4.94 | 9.6 |
| Gabon | 6.93 | 4.24 | 10.67 |
| Nicaragua | 6.88 | 4.92 | 9.65 |
| Andorra | 6.81 | 4.92 | 9.3 |
| Australia | 6.76 | 5.16 | 8.9 |
| Portugal | 6.76 | 4.85 | 9.54 |
| Spain | 6.73 | 4.87 | 9.17 |
| Nigeria | 6.73 | 4.38 | 9.8 |
| Grenada | 6.66 | 4.8 | 9.26 |
| Tokelau | 6.55 | 4.66 | 9.25 |
| Chile | 6.51 | 4.7 | 8.9 |
| United States of America | 6.47 | 4.97 | 8.38 |
| Niue | 6.44 | 4.6 | 8.96 |
| Jamaica | 6.41 | 4.64 | 9 |
| Lao People's Democratic Republic | 6.39 | 4.67 | 9 |
| Austria | 6.36 | 4.58 | 8.85 |
| Samoa | 6.33 | 4.52 | 8.82 |
| Costa Rica | 6.33 | 4.48 | 8.92 |
| Equatorial Guinea | 6.33 | 3.91 | 9.78 |
| United States Virgin Islands | 6.3 | 4.47 | 8.85 |
| United Republic of Tanzania | 6.29 | 4.04 | 9.45 |
| South Africa | 6.27 | 4.16 | 8.97 |
| Afghanistan | 6.27 | 4.34 | 9.04 |
| Cook Islands | 6.21 | 4.23 | 8.99 |
| Oman | 6.18 | 4.32 | 8.8 |
| Angola | 6.1 | 3.82 | 9.47 |
| Mongolia | 6.1 | 4.45 | 8.48 |
| Singapore | 6.08 | 4.37 | 8.37 |
| Bhutan | 6.07 | 4.21 | 8.83 |
| Ghana | 6.06 | 3.76 | 9.64 |
| Uganda | 6.06 | 3.81 | 9.33 |
| Bahrain | 6.03 | 4.41 | 8.46 |
| Gambia | 6.03 | 3.77 | 9.4 |
| Russian Federation | 6.02 | 3.95 | 9.19 |
| Monaco | 6 | 4.39 | 8.26 |
| Congo | 5.99 | 3.68 | 9.24 |
| Malawi | 5.99 | 3.85 | 8.97 |
| Djibouti | 5.98 | 3.82 | 9.2 |
| Ethiopia | 5.97 | 3.97 | 8.77 |
| Guam | 5.96 | 4.27 | 8.45 |
| Mozambique | 5.94 | 3.86 | 8.97 |
| India | 5.92 | 3.98 | 8.79 |
| Nepal | 5.91 | 4.09 | 8.71 |
| Fiji | 5.9 | 4.25 | 8.33 |
| Republic of Korea | 5.89 | 4.25 | 8.22 |
| Mauritania | 5.86 | 3.68 | 8.97 |
| Kyrgyzstan | 5.86 | 4.12 | 8.42 |
| Togo | 5.82 | 3.67 | 8.85 |
| Senegal | 5.8 | 3.62 | 9.05 |
| Sierra Leone | 5.8 | 3.65 | 8.95 |
| Zambia | 5.8 | 3.63 | 8.99 |
| Saint Kitts and Nevis | 5.8 | 4.17 | 8.01 |
| Albania | 5.79 | 4.05 | 8.21 |
| Saint Vincent and the Grenadines | 5.79 | 4.14 | 8.13 |
| Tajikistan | 5.77 | 4.03 | 8.43 |
| Namibia | 5.75 | 3.64 | 8.56 |
| Brazil | 5.74 | 3.85 | 8.29 |
| Belgium | 5.72 | 4.16 | 7.92 |
| Libya | 5.7 | 4.33 | 7.6 |
| Cameroon | 5.7 | 3.53 | 8.99 |
| Botswana | 5.67 | 3.56 | 8.59 |
| Germany | 5.65 | 4.04 | 7.92 |
| Sao Tome and Principe | 5.64 | 3.47 | 8.81 |
| Rwanda | 5.63 | 3.52 | 8.58 |
| Comoros | 5.6 | 3.59 | 8.48 |
| Benin | 5.59 | 3.52 | 8.76 |
| Burundi | 5.57 | 3.54 | 8.66 |
| Democratic Republic of the Congo | 5.56 | 3.4 | 8.67 |
| Guatemala | 5.55 | 3.91 | 7.95 |
| San Marino | 5.54 | 3.97 | 7.8 |
| Saint Lucia | 5.5 | 3.98 | 7.67 |
| Honduras | 5.46 | 3.89 | 7.75 |
| Yemen | 5.44 | 3.94 | 7.66 |
| Bolivia (Plurinational State of) | 5.42 | 3.79 | 8 |
| American Samoa | 5.41 | 3.85 | 7.82 |
| Côte d'Ivoire | 5.4 | 3.38 | 8.36 |
| Luxembourg | 5.38 | 3.95 | 7.3 |
| South Sudan | 5.34 | 3.4 | 8.16 |
| North Macedonia | 5.33 | 3.7 | 7.66 |
| Micronesia (Federated States of) | 5.32 | 3.81 | 7.53 |
| Tuvalu | 5.27 | 3.82 | 7.54 |
| Kiribati | 5.25 | 3.81 | 7.56 |
| Israel | 5.25 | 3.82 | 7.2 |
| Cyprus | 5.23 | 3.74 | 7.11 |
| Eritrea | 5.21 | 3.29 | 8 |
| Solomon Islands | 5.2 | 3.71 | 7.44 |
| Dominican Republic | 5.14 | 3.77 | 7.3 |
| Marshall Islands | 5.07 | 3.66 | 7.16 |
| Palau | 5.02 | 3.6 | 7.11 |
| Central African Republic | 5.01 | 3.09 | 7.85 |
| Guinea | 5.01 | 3.12 | 7.83 |
| Niger | 4.99 | 3.11 | 7.88 |
| Lesotho | 4.98 | 3.11 | 7.66 |
| Mali | 4.9 | 2.97 | 7.69 |
| Eswatini | 4.89 | 3.08 | 7.43 |
| Kazakhstan | 4.87 | 3.37 | 7.02 |
| Sudan | 4.86 | 3.47 | 6.85 |
| Burkina Faso | 4.83 | 3.01 | 7.58 |
| Somalia | 4.78 | 3.04 | 7.34 |
| Madagascar | 4.77 | 3 | 7.39 |
| Nauru | 4.76 | 3.46 | 6.77 |
| Greenland | 4.72 | 3.63 | 6.23 |
| Bahamas | 4.71 | 3.3 | 6.81 |
| Pakistan | 4.7 | 3.18 | 6.84 |
| Montenegro | 4.68 | 3.28 | 6.78 |
| Chad | 4.53 | 2.79 | 7.13 |
| Liberia | 4.52 | 2.88 | 6.87 |
| Vanuatu | 4.5 | 3.27 | 6.27 |
| Suriname | 4.5 | 3.15 | 6.39 |
| Belize | 4.45 | 3.16 | 6.34 |
| Papua New Guinea | 4.43 | 3.2 | 6.34 |
| Egypt | 4.38 | 2.77 | 6.77 |
| Zimbabwe | 4.36 | 2.71 | 6.59 |
| Haiti | 4.34 | 3.16 | 6.14 |
| Syrian Arab Republic | 4.34 | 2.97 | 6.29 |
| Dominica | 4.2 | 3.01 | 5.96 |
| New Zealand | 4.19 | 3.08 | 5.68 |
| Argentina | 4.08 | 2.81 | 5.93 |
| Morocco | 3.88 | 2.77 | 5.5 |
| Guyana | 3.72 | 2.57 | 5.46 |
| Uzbekistan | 3.58 | 2.42 | 5.44 |
| Guinea-Bissau | 3.42 | 2.15 | 5.19 |
| Brunei Darussalam | 2.91 | 2.02 | 4.15 |
| Azerbaijan | 2.64 | 1.82 | 3.82 |
| Georgia | 2.48 | 1.87 | 3.36 |
| Turkmenistan | 2.18 | 1.54 | 3.15 |
